# Adapting the SHARE Program for Use With Families Facing the Challenges of Chronic Illness

**DOI:** 10.1093/geroni/igaa057.2134

**Published:** 2020-12-16

**Authors:** Silvia Orsulic-Jeras, Carol Whitlatch

**Affiliations:** Benjamin Rose Institute on Aging, Cleveland, Ohio, United States

## Abstract

Advances in diagnostic procedures have helped to make diagnosing Alzheimer’s disease and other dementias more accurate and to occur earlier in the disease progression. For persons living with dementia and their family care partners, finding programs that meet their needs for support post diagnosis can be challenging. Likewise, for persons with chronic conditions, few programs exist which help care dyads to create a manageable plan of care that addresses each person’s concerns and fears. SHARE, (Support, Health, Activities, Resources, and Education), originally designed for dementia care partners, has shown positive outcomes for both members of the care partnership. This presentation describes the development of the six-session SHARE intervention, its implementation in community settings, and its current standing as an evidence-based program and product that has been commercialized. Discussion will also focus on adapting SHARE for use with chronic illness families, highlighting revisions to program procedures, materials, recruitment, and evaluation.

